# Why Educational Neuroscience Needs Educational and School Psychology to Effectively Translate Neuroscience to Educational Practice

**DOI:** 10.3389/fpsyg.2020.618449

**Published:** 2021-01-14

**Authors:** Gabrielle Wilcox, Laura M. Morett, Zachary Hawes, Eleanor J. Dommett

**Affiliations:** ^1^School and Applied Child Psychology, Werklund School of Education, University of Calgary, Calgary, AB, Canada; ^2^Hotchkiss Brain Institute, Cumming School of Medicine, University of Calgary, Calgary, AB, Canada; ^3^Mathison Centre for Mental Health Research & Education, University of Calgary, Calgary, AB, Canada; ^4^Alberta Children’s Hospital Research Institute, Calgary, AB, Canada; ^5^Department of Educational Studies in Psychology, Research Methodology, and Counseling, University of Alabama, Tuscaloosa, AL, United States; ^6^Ontario Institute for Studies in Education, University of Toronto, Toronto, ON, Canada; ^7^Institute of Psychiatry, Psychology and Neuroscience, King’s College London, London, United Kingdom

**Keywords:** educational neuroscience, school psychology, educational psychology, interdisciplinary, implementation

## Abstract

The emerging discipline of educational neuroscience stands at a crossroads between those who see great promise in integrating neuroscience and education and those who see the disciplinary divide as insurmountable. However, such tension is at least partly due to the hitherto predominance of philosophy and theory over the establishment of concrete mechanisms and agents of change. If educational neuroscience is to move forward and emerge as a distinct discipline in its own right, the traditional boundaries and methods must be bridged, and an infrastructure must be in place that allows for collaborative and productive exchange. In the present paper, we argue that school psychologists have the potential to fulfill this need and represent important agents of change in establishing better connections between research and practice. More specifically, we use the National Association of School Psychologists (NASP) (2020) Domains of Practice to highlight several areas where school psychology can actively support forging connections between neuroscience and educational practice. School psychologists represent untapped potential in their knowledge, skillset, and placement to serve a vital role in building the bridge between neuroscience and education.

## Introduction

Educational neuroscience has been growing as an area of research and practice over the last several decades. Due to the challenges of translating neuroscience into educational practice, there have been calls for educational psychology to serve as a translational bridge between the two fields (Bruer, 2006; Mason, 2009; Craig et al., 2020). Educational psychology training includes areas related to educational systems and practices; prevention, assessment, intervention, and progress monitoring; basic neuroscience and research understanding; and a strong foundation in cognitive constructs (e.g., attention, memory). Moreover, training in consultation can help school psychologists to bridge neuroscience and educational practice (Guli, 2005). Despite this skill set and these calls, educational psychology broadly and school psychology specifically have been relatively absent from educational neuroscience research and practice. In this paper, we discuss how educational and school psychologists can bridge neuroscience research and educational practice, helping to close the gap between them.

## Barriers to School Psychology Involvement in Translating Neuroscience Research Into Educational Practice

Although educational neuroscience was introduced over 30 years ago (Cruickshank, 1981; Fuller and Glendening, 1985), some have argued that educational neuroscience cannot be translated to education (Bruer, 1997; Bowers, 2016). However, since his initial assertion that the distance between neuroscience and education was too far to bridge, Bruer (2006) has noted that psychology can support the bridge building between the two fields. Howard-Jones et al. (2016) note that educational researchers use neuroscience to understand behavioral data. Neuroscience has impacted educational practice in several ways. For example, it has informed the mechanisms of dyslexia and interventions for dyslexia (Shaywitz and Shaywitz, 2008) and insights into how anxiety, attention, relationships, and sleep impact educational outcomes (Goswami, 2006; Carew and Magsamen, 2010).

Barriers to implementing neuroscientific research in education are well-established and include theoretical barriers such as the two disciplines having fundamentally different goals (descriptive vs. prescriptive) and scales of investigation (millisecond-level neurons firing vs. minutes-to-hours level conceptual change; Willingham, 2009; Devonshire and Dommett, 2010). To illustrate such incompatibility, it is worth asking “what counts as best practice” according to each discipline. Although there is no clear answer to this question, one can imagine a situation where best practice in teaching involves flexible, moment-to-moment adaptations and responses to the immediate environment and the needs of the students’ being taught. This is at odds with what many would consider evidence of best scientific practice; that is, the production of scientific knowledge that is replicable and reproducible. In the classroom, there is limited control, and practitioners must embrace complexity and the “blooming, buzzing confusion” of classroom learning (e.g., see Brown, 1992, p. 141). In the lab, one often tries to do away with complexity, simplifying the environment through utmost control. This highlights the need for implementation research by professions such as school psychology bridge effectively bridge this gap (Craig et al., 2020).

An additional superordinate theoretical barrier results from difficulties translating findings even when they serve relevant goals and are conducted at a suitable level of investigation. For example, it is unclear how the finding that inferior parietal sulcus subserves numerical processing can be leveraged to develop a curriculum that will improve student mathematical outcomes. It is suggested that this translational barrier can be overcome by changing the perspective through which the two fields are viewed. Churches et al. (2020) suggest that neuroscience can underpin education in the same way that biology underpins medicine, meaning that each field preserves its creativity but cannot operate against the law of the other field. This analogy is built on the fact that neuroscience is a natural science tasked with investigating the workings of the brain, much as biology understands the workings of the human body. By contrast, education aims to develop pedagogies and therefore has more in common with the manner in which medicine uses biology to ground research and practice in treatments, or the way architecture using physics.

School districts in close proximity to universities with school or educational psychology programs are more likely to have opportunities to collaborate on research related to translating educational neuroscience into practice. Additionally, well-funded school districts are more likely to have adequate staffing to allow school psychologists the time to consult with teachers to support translation of research into practice. As a result, students in schools with a greater research-to-practice gap, including those with significant populations of minority, Indigenous, and rural students, are less likely to benefit from these changes without intentional research attention and educational policies at state, provincial, or federal levels (Forman et al., 2013; Valdez et al., 2019).

Additional practical barriers to implementation that negatively impact effective translation of educational neuroscience to practice in schools include lack of support by administration and teachers, as well as limited training and technical support in implementing new practices, finances, and differing philosophical orientations, especially concerning research (Forman et al., 2013). Additionally, there are barriers to implementing new practices in schools, including demands of other role responsibilities and gaining administrator buy-in when there are competing demands (Kelly, 2012).

## What Educational Psychology Has to Offer Educational Neuroscience

Educational psychology is limited to research; thus, educational psychologists typically work in research and academic settings. School psychologists are involved in practice, typically working in school districts supporting students’ academic and social-emotional development and supporting teachers. They also work in universities as school psychology professors and contribute to school psychology related research (American Psychological Association (APA), n.d.). For the purposes of this paper, the terms are used interchangeably, as the research of both is relevant, and some countries do not distinguish between them. The National Association of School Psychologists (NASP) (2020) Domains of Practice serves as a framework for school psychologists’ knowledge and skills that can be used to support the translation of neuroscience into practice in schools. While all of these domains potentially support the translation of neuroscience into educational practice, we highlight the role of a few of the more prominent ones: data-based decision-making, academic interventions and instructional supports, and school-wide practices to promote learning.

## Data-Based Decision-Making and Research and Evidence-Based Practice

In recent decades, there has been a push to increase the rate of evidence-based practice (EBP) in a variety of fields. This has been challenging in many fields, but there are additional barriers in educational settings, and there is a significant research to practice gap resulting in low levels of EBP (Forman et al., 2013). Unfortunately, educational neuroscience is often ineffectively translated into educational practice, resulting in neuromyths—inaccurate understandings of neuroscientific literature that gain traction in discourse and practice (Organization for Economic Co-operation and Development (OECD), 2007; see Howard-Jones, 2017; Kim and Sankey, 2017, for discussions). They often arise from oversimplifications of complex findings to make them accessible to the public or due to difficulty in understanding neuroscientific information (Fischer et al., 2010). For example, brain images (McCabe and Castel, 2008) and neuroscientific language (Weisberg et al., 2008) interfere with readers’ ability to determine the accuracy of the reported findings. Unfortunately, entrepreneurs have also taken advantage of the power of neuroscientific language to sell products and services (Murphy et al., 2008). These neuromyths are perpetrated both by sources of variable repute (books, magazines, television, and websites) and sources that are typically deemed to be reputable (teachers, professors), which contributes to their intractability (Kim and Sankey, 2017). Teachers, who have frequently expressed interest in understanding educational neuroscience findings to better understand and support their students’ academic and social-emotional development, are left with few sources of vetted information (Goswami, 2006; Pickering and Howard-Jones, 2007; Hook and Farah, 2013).

School psychologists are trained in evaluating research as well as translating it into effective practice within school systems and thus can help to help fill this gap, aiding school administrators and teachers in determining the quality of evidence supporting programs and strategies purporting to be based upon educational neuroscience principles. School psychologists are trained in EBP in a variety of areas overlapping with educational neuroscience. Indeed, school psychology has been at the forefront of research informing EBP in many areas, including academics (e.g., Fuchs et al., 2017), emotional and behavioral development (e.g., Hoagwood et al., 2007), and support for students with disabilities (e.g., ADHD, ASD; Pfiffner et al., 2015; McClain et al., 2018). Additionally, school psychologists are trained in measuring outcomes at the individual, group, and system levels, making them well-placed to support translation of neuroscientific findings and to measure effectiveness of instructional changes. Recent research by Churches et al. (2020) has demonstrated that classroom teachers can lead randomized controlled trials and replication studies focused on neuroscience-informed hypotheses, contributing to a strong evidence base for practice through conducting research in a school environment. School psychologists are one of the few school personnel who have sufficient knowledge in experimental design to support teachers in this endeavor.

## Academic Interventions and Instructional Practices

School psychologists have been instrumental in developing academic interventions and instructional practices to improve student learning and outcomes in core domains including reading and mathematics (Bramlett et al., 2010). Training in educational neuroscience conveys key findings that can be leveraged to inform the development and refinement of interventions and practices, increasing their likelihood of demonstrating effectiveness in rigorous pre-implementation testing such as randomized controlled trials. For example, children with dyslexia, unlike their non-dyslexic peers, fail to recruit the dorsolateral prefrontal cortex during phonological awareness tasks (Kovelman et al., 2012). This finding suggests that interventions targeting executive function as well as phonological awareness may be more effective in improving the reading outcomes of dyslexic children than interventions targeting phonological awareness alone. Likewise, relative to typical peers, children with developmental dyscalculia demonstrate weaker functional activation in right intraparietal sulcus, insula, and inferior frontal lobe during a spatial working memory task as well as impaired working memory proficiency (Rotzer et al., 2009). This finding suggests the potential importance of targeting spatial working memory in mathematics interventions for children with dyscalculia. Indeed, converging evidence from the fields of both neuroscience and psychology indicate robust and reliable associations between spatial and mathematical processing (Mix and Cheng, 2012; Hawes et al., 2019; Hawes and Ansari, 2020). This provides yet another demonstration of how different levels of analysis (brain and behavior) can give rise to a richer understanding of domain-specific academic performance (e.g., mathematics) as well as the mechanisms that might underlie successful interventions. Most recently, researchers have leveraged this knowledge base to design both lab- and classroom-based training interventions to improve children’s spatial skills as well as their mathematics performance (e.g., see Cheng and Mix, 2014; Lowrie et al., 2017; Gilligan et al., 2019; but also see Hawes et al., 2015).

School psychologists, with their training in both research and practice, are uniquely situated to help school administrators and educators make evidence-informed decisions about which interventions to implement and which ones to potentially question and avoid. For example, many teachers currently endorse the effectiveness of using colored overlays to improve reading performance (Howard-Jones, 2014; Craig et al., 2020). With an understanding that the most common neurobiological cause of reading difficulties is weak phonemic awareness, an auditory process (Stanovich, 2009; Dehaene et al., 2010), teachers would be able to determine that using colored lenses is ineffective in addressing reading challenges. In light of their strong training in school-based research and assessment methods, school psychologists are well-poised to translate neuroscientific knowledge into evidence-based academic interventions and instructional practices that can be deployed to narrow the achievement gap between low- and high-achieving students.

Once academic interventions have been demonstrated effective in improving student outcomes via limited-scale rigorous pre-implementation testing, they can be implemented on a large scale via response to intervention (RTI; Fletcher and Vaughn, 2009). The RTI process, an area to which school psychology has contributed in research and practice, involves three tiers of intervention progressively increasing in intensity: core curriculum interventions and universal screening (Tier 1), small group supplementary interventions and instruction (Tier 2), and individualized intensive interventions (Tier 3). Students’ performance in response to each level of intervention, as assessed via curriculum-based measurement, determines whether students require the subsequent level of intervention. School psychologists are frequently involved in all aspects of the RTI process, including assessment administration and interpretation as well as intervention development and implementation, making them ideal translators of educational neuroscience findings into school practice and ideal collaborators in applied educational neuroscience research.

## School-Wide Practices to Promote Learning

Moving beyond domain-specific approaches (e.g., EBP specific to reading and math) to improving student outcomes, there exists an assortment of more general, school-wide approaches based on both behavioral and neuroscientific evidence to improve student outcomes and well-being. These principles of learning are based on decades of replicable and robust evidence (e.g., see Dunlosky et al., 2013). For example, there is substantial support from educational psychology literature on the effectiveness of practice testing (e.g., see Roediger and Butler, 2011), spaced/distributed learning (e.g., see Kang, 2016), and interleaved practice (Brunmair and Richter, 2019). Additionally, principles of effective learning have come to include the physiological conditions or requirements for learning to take place (Sigman et al., 2014). Recent research also provides new insights and details into the effects of nutrition, sleep, and exercise habits on learning, and critically, the underlying physiological and neural mechanisms at play (Curcio et al., 2006; Hillman et al., 2008; Sigman et al., 2014). Taken together, these principles of learning offer great promise and an encouraging starting place for school-wide initiatives that aim to use EBP to enhance student learning. School psychologists’ roles place them within schools, providing the relationships necessary to support effective implementation.

However, as discussed above, it is insufficient to generate a list of principles of learning and hope that educators and school administrators actively seek and integrate these findings into practice. Instead, intermediary actions are required (James,1899/2001). This is precisely where we see school psychologists, who are uniquely situated to connect research and practice, contributing. Below, we offer a practical example of how school psychologists might fulfill this role.

## Moving Forward: Connecting Research and Practice

As indicated previously, one way in which we might move forward is to draw inspiration from the relationship between biology and medicine, which has faced challenges similar to those faced by neuroscience and education. In much the same way that medical practice is not a biological experiment, classrooms are not neuroscience testing spaces. Additionally, given the huge variation in pupils and teachers, wide replication of research is needed to account for individual differences and to establish effectiveness in addition to efficacy. Consequently, there is a need to conduct real-world experiments encompassing key variations in educational systems and environments, akin to medical clinicians publishing their findings. The latter requires teachers, with the support of school psychologists, to be actively involved in research, something that is rarely found. The lack of teacher involvement in conducting and evaluating research is compounded by teacher training programs which provide minimal, if any, training on evaluating the quality of research.

It is arguably impossible to implement new training and research roles for all teachers; however, ensuring training and research structures are in place within the broader educational setting is important. One option is to adopt a research school approach, similar to teaching hospitals, in which school personnel are actively involved in designing and conducting experiments to provide a real-world evidence base for practice. A network of such schools would also support the replication of studies and use of meta-analysis, an approach which has been shown to be viable in principle with school psychologists as the primary in-house support for implementation (Thomas et al., 2019; Churches et al., 2020).

Another way in which we might move forward is by encouraging collaborations between academic school psychology and educational neuroscience programs. Collaborating programs could consider jointly appointing a liaison to serve as an intermediary between their researchers and school personnel to facilitate collaborative research and translation of research findings to curricula, pedagogical practices, and interventions. Moreover, such programs could also consider establishing outreach programs in which school psychology and educational neuroscience researchers meet regularly with school personnel, with school psychologists as key school-based liaisons. Doctoral students in school psychology would be ideal researchers in a program like this because they need to conduct research as part of their degree, and due to the nature of their training, they are often interested in applied research. This kind of partnership would provide a win-win, involving schools in research including determining research questions and providing school psychology students applied, collaborative research opportunities. For example, we (lead author) are conducting collaborative research initiated by a local school examining the impact of providing educational neuroscience professional development to teachers on both teachers and students.

Such programs would not only allow researchers to communicate their key findings to school personnel in lay terms with a focus on how they can be leveraged to improve student learning and outcomes, but also to collaborate with school personnel in developing research agendas addressing questions relevant to practice. As an adjunct to this school personnel-focused outreach, school psychology and educational neuroscience researchers could also provide periodic outreach within schools in which they teach both teachers and students about the brain and learning and provide opportunities for interested students and teachers to become involved in conducting research within schools or the lab. School psychologists are well-positioned to help coordinate these outreach activities and could serve as school-based liaisons, mediating between school personnel and academic school psychology and educational neuroscience researchers. School psychology practitioners have already demonstrated this skill in supporting school districts across the United States in understanding and implementing RTI to prevent school difficulties and interventing early to improve outcomes for students. Such liaison and outreach programs will help to establish the strong relationships between academic school psychology and educational neuroscience researchers and school personnel needed to conduct collaborative translational research benefiting both parties.

## Conclusion

Educational neuroscience has been criticized for having little impact on educational practice. The lack of concrete mechanisms bridging neuroscience and education has been a major contributor to this gap. In this paper, we have argued that school psychologists represent an untapped talent pool that can help fill this void. Educational and school psychologists possess the *bilingual* skills of understanding both basic neuroscience and education in addition to the psychological constructs relevant to both (Mason, 2009). Unfortunately, to date, school psychology has not played a prominent role in educational neuroscience. We argue that school psychology, as a field of research and practice, has the knowledge, the skillset, and the positioning to effectively contribute to building a bridge between education and neuroscience. Thus, school psychology has the potential to increase bi-directional collaboration between these fields, improving access to accurate information for teachers and, consequently, student outcomes.

## Data Availability Statement

The original contributions presented in the study are included in the article/supplementary material, further inquiries can be directed to the corresponding author/s.

## Author Contributions

GW outlined the idea. All authors equally contributed to the writing of the manuscript.

## Conflict of Interest

The authors declare that the research was conducted in the absence of any commercial or financial relationships that could be construed as a potential conflict of interest.
